# Dilated Cardiomyopathy as a Rare Presentation of Multisystem Inflammatory Syndrome in Children (MIS-C): A Case Report

**DOI:** 10.4314/ejhs.v33i6.20

**Published:** 2023-11

**Authors:** Vipulkumar V Gandhi, Komal Chopra

**Affiliations:** 1 Imperial Multispecialty Hospital, Chikhali, Pune, India; 2 Dr. D. Y. Patil Medical College & Research Centre, Pune, India

**Keywords:** Multisystem Inflammatory Syndrome in Children (MIS-C), Dilated cardiomyopathy, COVID-19, hyperimmune response

## Abstract

Multisystem inflammatory syndrome in children (MIS-C) is a rare post-infectious complication associated with COVID-19. This case report presents a detailed account of a paediatric patient diagnosed with MIS-C who developed dilated cardiomyopathy as a significant complication. This report aims to enhance our understanding of the rare potential cardiovascular implications of MIS-C and highlights the importance of prompt recognition and management.

## Case Presentation

A previously healthy 2-year-4-month-old boy presented to the Emergency Department with complaints of multiple episodes of vomiting and difficulty breathing over the last couple of days before admission. Parents also noticed cold extremities and excessive lethargy in the child. Physical examination revealed an acidotic breathing pattern with subcostal recessions. The child was febrile, profusely sweating, with a heart rate of 170 beats per minute, and non-recordable blood pressure with poor perfusion. Clinical examination showed that his peripheral pulses were non-palpable, and the central pulses were feeble. The first blood gas analysis on admission showed a pH of 7.16, a bicarbonate of 10.2 with a base excess of -18.3, and a high lactate value of 8.7.

**Figure 1:**
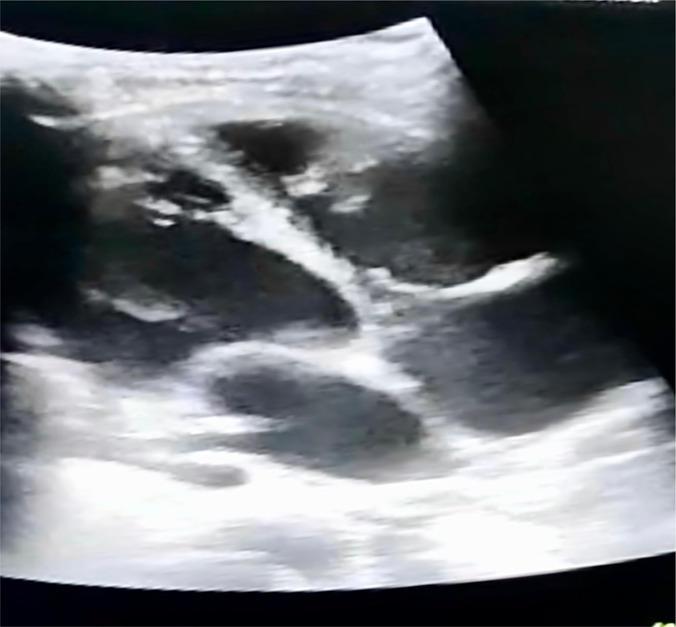
Bedside Echocardiogram: 4 chamber view showing BV dilatation on day 1 of admission

Considering a provisional diagnosis of cardiogenic shock; bedside echocardiography was performed, revealing a dilated biventricular pattern with predominant left ventricular dilatation and mitral regurgitation. There was global hypokinesia and a distended inferior vena cava with an underlying ejection fraction of 10% (Figure 1).

Given the underlying severe cardiogenic shock, the child was electively intubated and ventilated and started on inotropic support after securing central venous access. He received a cautious fluid bolus of 20 ml/kg of crystalloids in small aliquots. Blood investigations showed markedly elevated inflammatory markers (neutrophilic leucocytosis, C-reactive protein, lactate dehydrogenase, serum ferritin, procalcitonin, fibrinogen levels, and D-dimer levels) along with deranged cardiac biomarkers (Troponin T and CK-MB). There was evidence of a myocardial strain pattern with biventricular dilatation and a normal PR interval on the electrocardiogram.

**Figure 2:**
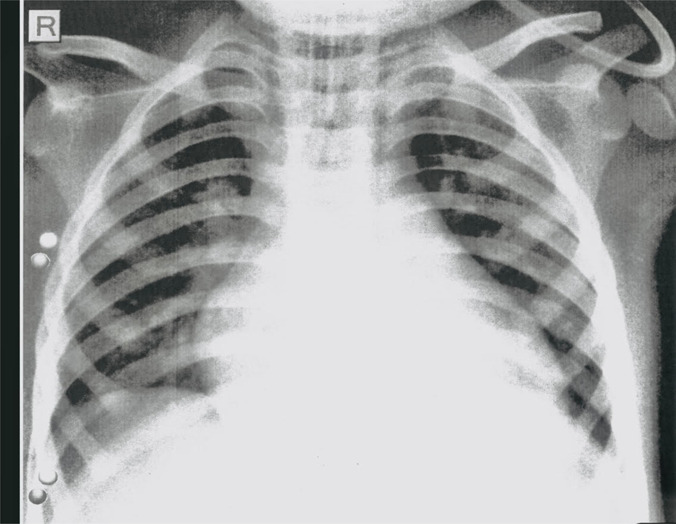
Chest X-ray: Cardiomegaly with bilateral plethoric lung fields

The chest X-ray showed cardiomegaly with bilateral plethoric lung fields (Figure 2). Blood cultures and a viral PCR panel for respiratory infection remained negative. On further inquiry, it was found that is family had a recent COVID-19 infection, and the child had positive SARS-CoV-2 IgG antibodies, confirming the diagnosis of MIS-C. To rule out other differential diagnoses, the cardiomyopathy screening panel, including serum calcium levels, vitamin D, thyroid function test, creatinine kinase level, metabolic screen, and cardiomyopathy viral panel, was documented as negative. Other lab parameters showed liver dysfunction with significantly elevated alanine transaminase (ALT) levels but normal blood ammonia and clotting profile. He also had acute kidney injury with abnormal serum creatinine levels, which peaked on day 2 of admission and gradually normalized during the Paediatric Intensive Care Unit (PICU) course.

In the PICU, on day 1 of admission, hemodynamic stability was achieved after receiving fluid optimization, inotropic support, and mechanical ventilation. Initially, the child received broad-spectrum antibiotics, which were later discontinued with sterile blood culture reports. His metabolic derangement was corrected with sodium bicarbonate for severe metabolic acidosis. As the child had persistent hypotension despite multiple inotropes, catecholamine-resistant shock was suspected, and he was started on a stress dose of IV hydrocortisone.

On day 2 of admission, after confirming the diagnosis of MIS-C through clinical presentation and laboratory evaluation, he was started on immunomodulators, such as pulse-dose methylprednisolone and a course of intravenous immunoglobulin (IVIG) at 2 g/kg. The child had a fever for the first few days, which subsided after treatment with the IVIG course. The congestive cardiac failure was managed with regular diuretics, restricted fluids, and inotropic support. Along with immunomodulators, the child received low molecular weight heparin (LMWH) and aspirin according to standard MIS-C treatment guidelines.

Serial echocardiograms demonstrated marginal improvement in cardiac ejection fraction up to 30%. The improvement in cardiac function led to successful weaning from inotropic support by day 6 of admission. On day 9 of admission, he was extubated with high-flow nasal cannula support. His inflammatory markers and end-organ parameters gradually improved and normalized during the first week of PICU management. After a prolonged hospital stay, the child was discharged on anti-failure medications, including regular diuretics and oral Enalapril (an ACE inhibitor), in addition to regular low-dose aspirin. He was closely followed up for ongoing cardiology care. His follow-up ECHO showed marginal improvement in ejection fraction up to 40% with no coronary involvement on the 3- and 6-month follow-up visits post-discharge from the PICU.

## Discussion

Although the acute presentation of a COVID-19 infection in the paediatric population may not be too dramatic, MIS-C, a post-COVID complication, has created various management challenges during the pandemic (1,2). The management of MIS-C requires a different treatment approach as it presents a spectrum of severity and multi-organ dysfunction (1,3). Dilated cardiomyopathy (DCM) has emerged as one of the rare presentations of MIS-C. DCM is characterized by left ventricle dilation and reduced systolic function, leading to heart failure and, later, multiple organ failure. While DCM can be caused by various secondary factors, such as myocarditis, arrhythmias, hypothyroidism, severe anaemia, and connective tissue disorders in children; primary or idiopathic DCM is considered in almost 70% of the patients where the causative agents remain elusive despite extensive evaluation. Some retrospective studies demonstrated the TTN gene mutation in 25% of familial cases of idiopathic DCM and 18% of sporadic cases. However, due to financial constraints, we did not conduct the DCM genetic mutation panel in our case.

A review article by Alsaied et al. highlighted various cardiac manifestations of MIS-C, including ventricular dysfunction secondary to myocarditis, coronary artery dilatation or aneurysm, arrhythmias, conduction abnormalities, and more rarely, pericarditis and valvulitis (4). This study showed left ventricular dysfunction in nearly half of the patients with MIS-C. However, none of the case series reviewed in this study documented DCM, as seen in our patient. In our case, the child had DCM in the context of a recent positive history of COVID-19 infection in the family, along with a positive COVID-19 antibody test. Appropriate diagnostic screening for DCM failed to identify a known secondary causative agent. This is the reason we propose DCM as a rare possible presentation of MIS-C. With current technological advancements, the American Heart Association (AHA) has recommended cardiac magnetic resonance (CMR) studies as the gold standard for diagnosing myocarditis in children. It has been observed that parametric mapping of CMR not only aided in diagnosing acute and chronic myocarditis in children but also in differentiating myocarditis-related DCM from the idiopathic entity during the COVID-19 pandemic. However, due to funding limitations, we could not perform the CMR study in our case to rule out myocarditis-related DCM from the idiopathic entity.

With this case report, we recognize the possibility of incidental findings of positive COVID-19 antibody reports in the setting of an independently at-risk child developing DCM from alternative pathophysiological mechanisms. However, the clinical presentation of cardiogenic shock secondary to DCM confirmed on echocardiography, associated with raised inflammatory markers seen in MIS-C, along with positive COVID antibodies and an extensive negative DCM workup, strengthens our impression of potential MIS-C-related pathogenesis.

The pathogenesis of MIS-C-related cardiomyopathy remains unclear, with hypotheses suggesting a combination of direct viral myocardial damage, immune dysregulation, and a cytokine storm through ACE2 receptors expressed on myocytes and vascular endothelial cells (5). Additionally, the stress response related to shock and hypoxemia, causing a surge in catecholamines and Cortisol, can contribute to DCM pathogenesis. We postulate that a combination of these factors is responsible for myocyte injury leading to DCM. This case describes the challenges of managing dilated cardiomyopathy in the context of MIS-C. The initial phase of supportive management focused on hemodynamic stability during cardiogenic shock, followed by a thorough clinical workup to confirm the diagnosis and rule out treatable secondary causes. Once the diagnosis was confirmed after ruling out other possibilities, management focused on definitive treatment, including immunomodulation through pulse-dose steroids and IVIG for MIS-C and heart failure management for DCM. Regular follow-up of inflammatory marker trends, serial cardiac biomarkers, and echocardiography helps clinicians skilfully titrate the treatment.

In conclusion, MIS-C is an established clinical entity associated with COVID-19, and dilated cardiomyopathy represents a significant and unusual potential complication. Early recognition, multidisciplinary management, and close follow-up are crucial for optimizing outcomes in paediatric patients with MIS-C-related cardiomyopathy. This case report contributes to our understanding of the disease's clinical spectrum and emphasizes the need for ongoing research to elucidate its underlying mechanisms.
